# New Method for Simultaneous Determination of Microcystins and Cylindrospermopsin in Vegetable Matrices by SPE-UPLC-MS/MS

**DOI:** 10.3390/toxins10100406

**Published:** 2018-10-08

**Authors:** Leticia Díez-Quijada, Remedios Guzmán-Guillén, Ana I. Prieto Ortega, María Llana-Ruíz-Cabello, Alexandre Campos, Vítor Vasconcelos, Ángeles Jos, Ana M. Cameán

**Affiliations:** 1 Area of Toxicology, Faculty of Pharmacy, University of Sevilla, C/Profesor García González 2, 41012 Sevilla, Spain; ldiezquijada@us.es (L.D.-Q.); anaprieto@us.es (A.I.P.O.); mllana@us.es (M.L.-R.-C.); angelesjos@us.es (A.J.); camean@us.es (A.M.C.); 2 CIIMAR/CIMAR—Interdisciplinary Centre of Marine and Environmental Research, University of Porto, Terminal de Cruzeiros do Porto de leixões, Av General Norton de Matos, 4450-208 Matosinhos, Portugal; amoclclix@gmail.com (A.C.); vmvascon@fc.up.pt (V.V.); 3 Faculty of Sciences, University of Porto, Rua do Campo Alegre, 4169-007 Porto, Portugal

**Keywords:** microcystins, cylindrospermopsin, method validation, UPLC-MS/MS, lettuce

## Abstract

Cyanotoxins are a large group of noxious metabolites with different chemical structure and mechanisms of action, with a worldwide distribution, producing effects in animals, humans, and crop plants. When cyanotoxin-contaminated waters are used for the irrigation of edible vegetables, humans can be in contact with these toxins through the food chain. In this work, a method for the simultaneous detection of Microcystin-LR (MC-LR), Microcystin-RR (MC-RR), Microcystin-YR (MC-YR), and Cylindrospermopsin (CYN) in lettuce has been optimized and validated, using a dual solid phase extraction (SPE) system for toxin extraction and ultra-performance liquid chromatography-tandem mass spectrometry (UPLC-MS/MS) for analysis. Results showed linear ranges (5–50 ng g^−1^ f.w.), low values for limit of detection (LOD) (0.06–0.42 ng g^−1^ f.w.), and limit of quantification (LOQ) (0.16–0.91 ng g^−1^ f.w.), acceptable recoveries (41–93%), and %RSD_IP_ values for the four toxins. The method proved to be robust for the three variables tested. Finally, it was successfully applied to detect these cyanotoxins in edible vegetables exposed to cyanobacterial extracts under laboratory conditions, and it could be useful for monitoring these toxins in edible vegetables for better exposure estimation in terms of risk assessment.

## 1. Introduction

Eutrophication and climate change may promote the proliferation and expansion of harmful cyanobacterial blooms in freshwater, estuarine, and marine ecosystems [1]. An increasing number of cyanobacteria can produce toxic metabolites named cyanotoxins [2], which comprise a large variety of compounds with various structural and physicochemical properties [3]. Among these cyanotoxins, microcystins (MCs) and cylindrospermopsin (CYN) are amongst the most studied because of their widespread distribution. MCs, mainly produced by *Microcystis*, have the common cyclic heptapeptidic structure of cyclo(-d-Ala-l-X-d-MeAsp-l-Z-Adda-d-Glu-Mdha), in which X and Z are variable l-amino acids that give the name to the molecule [4]. Because of these modifications, and the methylation/desmethylation of several functional groups, more than 100 MC variants have been reported to date [5]. Hence, the most common congeners are MC-LR, MC-RR, and MC-YR, resulting from the presence of the L-form of leucine (L), arginine (R), or tyrosine (Y) in position 2, and R in position 4 [6]. On the other hand, CYN is an alkaloid consisting of a tricyclic guanidine moiety combined with hidroxymethiluracil, which can be produced by *Cylindrospermopsis raciborskii* and *Chrysosporum ovalisporum* [7]. Concerning the mechanism of action, MCs are hepatotoxins and tumor promoters due to their strong potent inhibition of protein phosphatases and the effect on cell signaling pathways [4]. CYN is well known by its inhibition of protein and glutathione synthesis, induction of oxidative stress, and cytochrome P450 seems to mediate its toxicity; its pro-genotoxic activity has also been studied [6,8]. Although these toxic effects on animals are well documented, fewer studies have focused on their effects on vegetables, either in leaves, roots, and stems, such as oxidative stress, alterations in growth, germination, and development, and in mineral and vitamin contents [4,9,10,11,12,13,14,15,16].

It is important to take in mind that these cyanotoxins are not produced alone and isolated in aquatic environments, since a coincidence in time and space of cyanobacterial blooms can produce several cyanotoxins in the same freshwater and marine ecosystem and the same cyanobacteria species can produce different toxins also. In surface waters used as an irrigation source, total MCs concentrations from 50 µg L^−1^ up to 6500 µg L^−1^ have been reported [1] and CYN environmental concentrations in surface waters ranged from 1 to 800 µg L^−1^ [17,18,19,20,21]. Thus, humans may be orally exposed to cyanotoxins by drinking contaminated water, through the consumption of cyanotoxin-containing freshwater fish, crops, and food supplements, or by ingesting water during recreational activities [6].

Indeed, edible vegetables may accumulate MCs in the range 1.03–2352.2 µg kg^−1^ d.w. [4,10,12,13,14,15,22,23] and CYN in the range 2.71–49,000 µg kg^−1^ f.w. [9,10,11,24] by direct contact with contaminated irrigation water, which represents an additional risk to public health. The data available in the literature show provisional Tolerable Daily Intakes (TDI) of 0.04 µg kg^−1^ of body weight (b.w.) for MCs [25] and 0.03 µg kg^−1^ b.w. for CYN [26]. The upper limit in drinking water of 1 µg L^−1^ was set for MC-LR [25] and, although there is still no legislation regarding CYN, the same value has been proposed for this toxin [26]. However, although no limits have been established for these toxins in edible vegetables, it would be of interest to have adequate tools for their accurate determination in these matrices.

Mass spectrometry (MS) techniques are a powerful tool for the analysis of biotoxins [27], and it has been employed in the case of MCs detection in natural blooms, cyanobacterial strains, fish, and other biological samples [28,29,30]. Specifically, ultra-performance liquid chromatography-tandem mass spectrometry (UPLC-MS/MS) allows excellent specificity and sensitivity for cyanotoxins’ detection and quantification in waters and in more complicated matrices, becoming the preferred technique for cyanotoxin analysis [16,31,32,33,34,35,36,37]. Previous studies were carried out in our laboratory for the determination of CYN in water [38], fish [39], and vegetables [40]. However, the separate isolation and identification of toxins belonging to each discrete class is laborious, expensive, and time consuming. Solid phase extraction (SPE) allows the cleaning of the sample for recovery and extraction of different analytes; however, the use of the same type of SPE cartridge is normally not adequate for the recovery of different cyanotoxins due to their differential physicochemical characteristics. Therefore, a multitoxin analytical approach is necessary for the simultaneous screening, detection, identification, and quantification of these toxins at low concentration levels [3], and in complex matrices, such as food items, following the recommendations of the European Food Safety Authority (EFSA) [41]. Different analytical methods for the simultaneous determination of cyanotoxins in waters are available, with [3,13,33,42,43] or without SPE [2,34,44,45]. By contrast, studies carried out to determine cyanotoxins simultaneously in vegetables are scarcer, and have not been validated. In addition, some of them have been performed with Enzyme-Linked ImmunoSorbent Assay (ELISA) [10], or have not looked at CYN [4,46,47]. For these reasons, and considering that MCs and CYN may coexist in natural environments, the aim of this work was to develop and validate a simple, sensitive, and robust analytical method for the simultaneous detection of the most common congeners of MCs (MC-LR, MC-RR, and MC-YR) and CYN in edible vegetables, such as lettuce, in a single analysis. The proposed method employed a dual SPE cartridge system in combination with UPLC-MS/MS, and has been optimized and tested according to international guidelines [48,49,50,51]. The present method has been designed to prove its applicability for the simultaneous analysis of these cyanotoxins in vegetable samples intended for human consumption.

## 2. Results and Discussion

### 2.1. Setup of the UPLC-MS/MS

Before assessing the effectiveness of the MC and CYN extraction method, the UPLC-MS/MS system was set up for this purpose. The transitions employed for MC-LR were 996.5/135.0, 996.5/213.1, and 996.5/996.5; for MC-RR: 520.2/135.0 and 1039.5/135.0; for MC-YR: 1046.5/135.0, 1046.5/213.0, and 1046.5/1046.5; and, finally, for CYN: 416.2/194.0 and 416.2/176.0; choosing the first ones for quantitation and the others as confirmatory, for each toxin. With respect to MC-LR, MC-RR, and MC-YR, the signals at *m*/*z* 135 and 213 were the main product ions for these MCs. The transition with *m*/*z* 520.2 corresponds to the double-charged protonated molecular ion, [M + 2H]^2+^. The product ion at *m*/*z* 135 was identified as the [phenyl-CH_2_-CH(OCH_3_)]^+^ ion formed by the rupture of the Adda residue between C-8 and C-9 and the *m*/*z* 213 was [Glu-Mdha + H^+^] lined up by the rupture of the α-linked glutamic (d-Glu) acid and *N-*methyldehydroalanine (Mdha) residue [4,52].

Regarding CYN, the signals at *m*/*z* 194 and 176 correspond to the loss of SO_3_ and H_2_O from the fragment ion at *m*/*z* 274, which represents the loss of the [6-(2-hydroxi-4-oxo-3-hydropyrimidyl)] hydroxymethinyl moiety from the CYN structure [53].

### 2.2. Calibration Study

**Figure 1:**
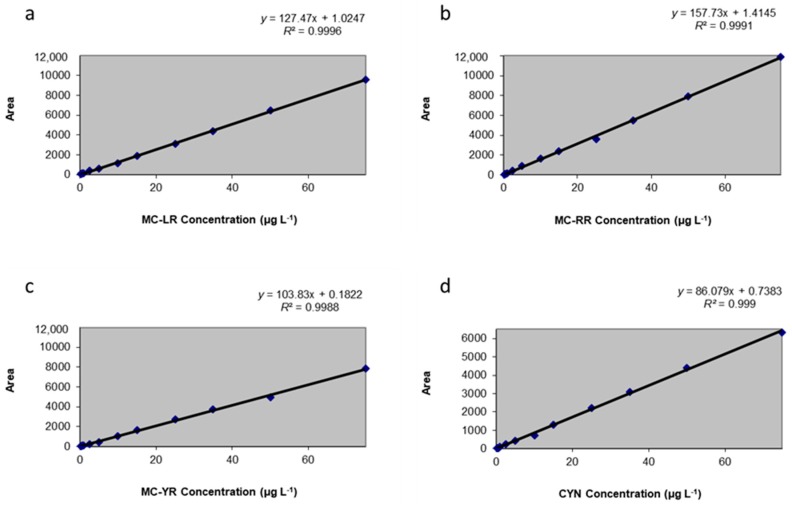
Calibration curves obtained for (**a**) Microcystin-LR (MC-LR); (**b**) MC-RR; (**c**) MC-YR; and (**d**) Cylindrospermopsin (CYN) in lettuce.

To perform a calibration study, it is necessary to subject known amounts of the quantity to the measurement process, monitoring the measurement response over the expected working range [51]. The reply as a function of the concentration were calculated from MC-LR, MC-RR, MC-YR, and CYN standards prepared in fresh lettuce leaves extracts and were measured by four 12-point calibration curves with a linear range within 0.2–75 µg toxins L^−1^ (equal to 0.2–75 ng toxins g^−1^ f.w. lettuce). Twelve points were required for an accurate linearity considering the low working concentrations in the present study. The regression equations obtained were (a) *y*: 127.47*x* + 1.0247 (*r*^2^ = 0.9996) for MC-LR; (b) *y*: 157.73*x* + 1.4145 (*r*^2^ = 0.9991) for MC-RR; (c) *y*: 103.83*x* + 0.1822 (*r*^2^ = 0.9988), for MC-YR; and (d) *y*: 86.079*x* + 0.7383 (*r*^2^ = 0.9999) for CYN (Figure 1).

#### 2.2.1. Linearity and Goodness of the Fit

**Figure 2:**
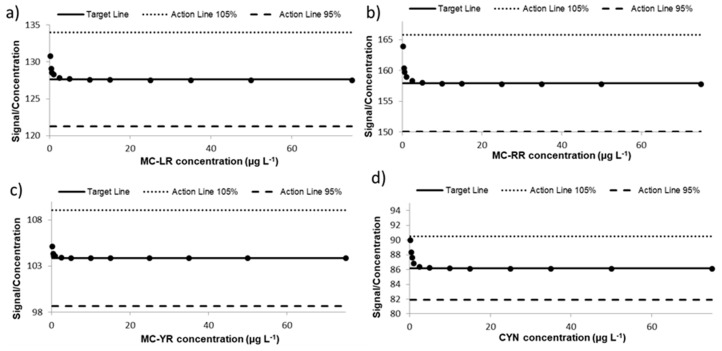
Response linearity in lettuce (Huber plot) for (**a**) MC-LR; (**b**) MC-RR; (**c**) MC-YR; and (**d**) CYN.

Linearity of an analytical method with regards to the analysis of a number of samples varying in analyte concentrations followed by regression statistics were performed [51]. Twelve different concentrations of the mixture solution containing MCs and CYN were spiked to blank extracts of lettuce leaves (*n* = 3) and the samples were submitted to the present validated method. According to Huber et al. [54] the calibration plot, which represents the signal response/analyte concentration relationship versus analyte concentrations, was assessed by replicate analysis (*n* = 3) of the extracts at 0.2–75 µg toxins L^−1^. The target line in Figure 2 represents the median signal/concentration calculated for all the concentrations evaluated. The results obtained for each concentration assayed are within the median value ±5% for MC-LR, MC-RR, MC-YR, and CYN, demonstrating the linear range of the proposed method.

With the same signal data, the goodness of the fit was calculated. A lack of the fit *F* ratio was obtained using adequate analysis of variance (ANOVA) of the regression lines. The results showed that *F* ratio data were lower than the tabulated value for the corresponding degrees of freedom in this study (<19.4): 0.96 for MC-LR, 1.88 for MC-RR, 0.75 for MC-YR, and 0.76 for CYN, showing linear calibration functions for all toxins.

#### 2.2.2. Sensitivity

For method validation, it is usually enough to give a sign of the level at which detection becomes problematic and quantification is adequate in terms of the precision, repeatability, and trueness. For this objective, the limits of detection (LOD) and quantification (LOQ) were determined with 10 independent samples, according to the equation, Y_LOD/LOQ_ = Y_blank_ + nS_blanck_, where Y_blank_ and S_blank_ are the average value of the blank signals and its corresponding standard deviation, and *n* is a constant value (3 for LOD and 10 for LOQ). Afterwards, LOD and LOQ values were converted in concentration using the calibration functions obtained previously. The LOD and LOQ values are presented in Table 1. Both the LOD and LOQ were lower than the guideline value of 1 µg L^−1^ proposed for MCs by WHO [25] and for CYN by Humpage and Falconer [26]. These results were lower or of the same order than those reported by Li et al. [4] in different vegetable matrices. The LOQ presented in the present work were in the lower limit of the ranges reported by those authors for MC-LR, -RR, and -YR in all the vegetable matrices. However, our results could not be compared with the same matrix (lettuce), in particular because these authors did not report the values for each matrix individually. Moreover, the present work reports for the first time, adequate LOD and LOQ levels for CYN in vegetables. More recently, Manubolu et al. [55] optimized an extraction method for MC-LR and MC-RR in different matrices, including vegetables. Nevertheless, the study conducted by these authors has two important limitations: The applicability of the method for the extraction of other MC congeners, and the quantification of the toxins at low concentrations (reported LOD < 26 ng g^−1^ f.w. and LOQ < 72 ng g^−1^ f.w.). Both analytical parameters have been improved in the present study. Furthermore, in the present multitoxin method that permits the simultaneous analysis of MCs variants and CYN, it presents some advantages over other methods that determine MCs or CYN individually, such as saving time, samples, materials, and resources, and diminishing solvent volumes, which could represent less environmental pollution.

#### 2.2.3. Matrix Effects

The matrix effect impacts the quantitation greatly and it depends on various factors, such as the properties of the analyte and the composition and amount of matrix [4]. Mild or no matrix effects simplifies calibration enormously if the calibration standards can be prepared as simple solutions of the analyte without matrix modifiers. For this, it is necessary to evaluate the effects of a possible general matrix mismatch in the validation process previously [56]. The lettuce matrix effect was studied for the four cyanotoxins following the recommendations of Li et al. [4]. The results showed matrix effect values of 4.23% (MC-LR), 17.17% (MC-RR), −12.32% (MC-YR), and −11.40% (CYN). Effects are considered mild (|10| < ME < |20|) for MC-RR, MC-YR, and CYN, or it can even be ignored (|10| < ME < |10|) in the case of MC-LR. Similar results were obtained by Li et al. [4] in the lettuce matrix for these three MCs (range from −13% to −5%); however, the present study shows for the first time that the presence of a new analyte in the mixture (CYN) does not increase the matrix effect.

### 2.3. Accuracy Study

For method validation, the accuracy of results is studied by considering both systematic and random errors. Thus, accuracy is analyzed as an entity with two components, such as trueness and precision [48,57].

#### 2.3.1. Precision

Precision is a measure of the closeness of agreement between mutually independent measurement results obtained under specified conditions and it is generally dependent on the analyte concentration [51]. According to the International Conference on Harmonisation Guidelines (ICH) [58], the measure of precision includes three concepts: Repeatability, intermediate precision, and reproducibility. Repeatability indicates the variability when measurements are carried out by a single analyst with the same material, method, and equipment over a short period of time. On the other hand, intermediate precision assesses the variation in results when measurements are made with the same material, method, and in the same laboratory over an extended period, and therefore represents more variability than repeatability. Otherwise, reproducibility represents the variability in results when measurements are made in different laboratories [51].

The values of repeatability (within-day and between-day, S_w_ and S_B_), intermediate precision (intralaboratory reproducibility, S_IP_), and S_IP_ relative standard deviations (%RSD_IP_) were calculated analyzing three replicates of lettuce leaf extracts spiked with the standard mixture solution containing MCs and CYN at different concentrations (5, 20, and 50 µg L^−1^) on the same day, following the ICH guidelines, and over a period of three consecutive days. Considering three different days as the main source of variation, the estimations of these precision parameters were obtained by performing an analysis of variance (ANOVA) for each validation standard according to González and Herrador [48] and González et al. [49]. The relative standard deviations (%RSD_IP_) obtained for each toxin were compared to the expected values issued by the AOAC Peer Verified Methods Program [49,50,54] for the different concentrations assayed (16–22% for 5 µg L^−1^ or 8–16% for 20 and 50 µg L^−1^). For all studied toxins, the %RSD_IP_ values presented were lower or of the same order than RSD_AOAC_ tabulated values at the three concentration levels assayed, so that the proposed method can be considered as precise. The results are shown in Table 1.

#### 2.3.2. Recovery

**Figure 3:**
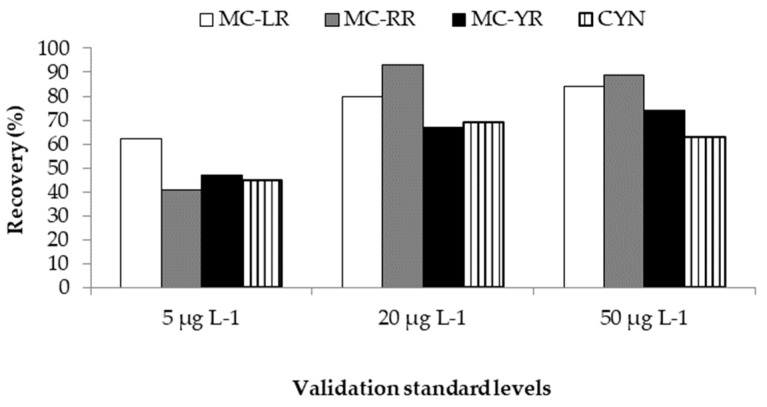
Recovery percentages for the three validation standards and cyanotoxins assayed.

Trueness is the closeness of agreement between a test result and the accepted reference value of the property being measured and it can be investigated by spiking and recovery [56]. Total recovery for any validation method is defined as the ratio between the observed estimation of the validation standard concentrations and the true value, T, expressed as a percentage or fraction [38]. The recoveries obtained in the present study were in the range of 62–84% (MC-LR), 41–93% (MC-RR), 47–74% (MC-YR), and 45–69% (CYN) (Figure 3). The adequacy of these results was checked with the recovery values accepted for each concentration range, which are 40–115% for 5 µg L^−1^ and 60–110% for 20 and 50 µg L^−1^ [50]. As can be observed in Figure 3, MC-LR showed the best recoveries at lower concentrations (5 µg L^−1^), whereas at higher concentrations (20 and 50 µg L^−1^), it seems that better recoveries are obtained for MC-RR followed by MC-LR. In general, slightly better results have been obtained for MCs compared to CYN. Previously, high recoveries (104% for 20 µg L^−1^) were shown for CYN in a validated method for determination of this toxin in lettuce [40]; however, in the present work, CYN recovery percentages resulted in compromised favor of the extraction and detection of more hydrophobic cyanotoxins, such as MCs.

Similar recoveries were obtained by Li et al. [4] for MCs (-LR and -RR), although these authors showed a better recovery for MC-YR. However, our study also includes the detection of another cyanotoxin (CYN), with acceptable recovery levels obtained, and which has not been included in the work from those authors. Similarly, other authors have also shown very good recoveries for MC-LR and -RR in lettuce [55]; this could be explained by two reasons: The low number of toxins analysed and the high concentrations they employed for spiking the samples (250 and 1000 ng g^−1^ f.w.) in comparison with ours (5–50 ng g^−1^ f.w.).

### 2.4. Robustness

The robustness, also called ruggedness, of an analytical method represents the resistance of the results to change after minor deviations are made in the experimental conditions described in the procedure. Thus, it is tested by deliberately introducing small changes to the procedure and examining the effect on the results [56]. The *t* values obtained for each cyanotoxin were compared with the 95% confidence level two-tailed tabulated value (*t*_tab_ = 2.306) corresponding with eight degrees of freedom obtained in the present study (Table 2). All the *t* values obtained were less than 2.306, so the present validated method can be considered as robust against the three different factors assayed at 20 µg L^−1^ for, MC-LR, MC-RR, MC-YR, and CYN. Although this is an important parameter to consider when validating analytical methods [51], as far as we know, no studies have validated robust methods for various cyanotoxins in any matrix, including vegetables.

### 2.5. Application to Real Samples: Edible Vegetables Exposed to MC and CYN-Producing Extracts

**Figure 4:**
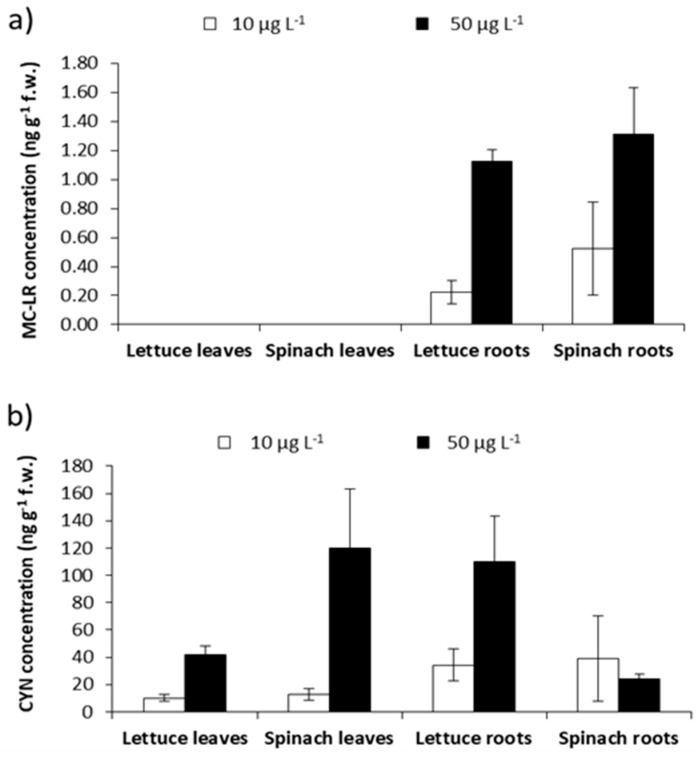
(**a**) MC-LR and (**b**) CYN concentrations detected in the leaves and roots of lettuce and spinach exposed to cyanobacterial extracts under laboratory conditions.

The optimized and validated method was applied for the detection and quantification of the main variants of MCs (MC-LR, -RR, and -YR) and CYN in lettuce and spinach, as described in Section 4.5. LOD and LOQ levels from the proposed method permit the detection of MC-LR and CYN in these samples (Figure 4). No MC congeners were detected in the leaves of both vegetables analyzed, only low MC-LR levels were detected (0.22–1.31 ng g^−1^ f.w.) in roots, whereas CYN concentrations in the leaves ranged between 10–120 ng g^−1^ f.w. and in roots, between 24–110 ng g^−1^ f.w.

## 3. Conclusions

For the first time, a method for the simultaneous detection of MCs (MC-LR, -RR, and -YR) and CYN has been optimized and validated in lettuce (*L. sativa*) by SPE-UPLC-MS/MS, showing acceptable linearity, sensitivity, precision, recovery, and robustness for all toxins. This method has been successfully applied to real vegetable samples intended for human consumption. Due to the simultaneous presence of different cyanotoxins in the environment, it is indispensable to have adequate validated methods for their accurate detection in a more realistic exposure scenario in terms of health risk assessment.

## 4. Materials and Methods

### 4.1. Chemicals and Reagents

Three congeners of MCs (MC-LR, MC-RR, and MC-YR) (99% purity) and Cylindrospermopsin standard (95% purity) were purchased from Enzo Life Sciences (Lausen, Switzerland). Deionized water (18.2 MΩ cm resistivity) was obtained from a Milli-Q water purification system (Millipore, Bedford, MA, USA). HPLC-grade methanol, dichloromethane (DCM), formic acid (FA), acetonitrile, and sodium hydroxide (NaOH) were supplied by Merck (Darmstadt, Germany). For the SPE, C18 cartridges were Bakerbond^®^ (500 mg, 6 mL), purchased from Dicsa (Andalucía, España), and graphitized carbon cartridges were BOND ELUT^®^ (500 mg, 6 mL), supplied by Agilent Technologies (Amstelveen, The Netherlands). For UHPLC–MS/MS analyses, reagents were of LC–MS grade: Water and acetonitrile were supplied by VWR International (Fontenay-sous-Bois, France) and formic acid by Fluka (Steinheim, Germany). A standard multitoxin solution containing 100 µg L^−1^ of each cyanotoxin (MC-LR, MC-RR, MC-YR, and CYN) was prepared in 20% MeOH to be further diluted to three different concentrations (5, 20, and 50 µg L^−1^), used as working solutions. Lettuce control samples (without toxins) were obtained from a local supermarket, ready for human consumption.

### 4.2. Toxin Extraction from Lettuce Leaves and SPE

Considering previous experiments in which the lyophilization process did not affect the recovery of toxins [40], the addition of toxins to the lettuce leaves (fortification process before extraction) was performed with fresh weight lettuce and then they were lyophilized. First, before testing the extraction efficiency for the four cyanotoxins, the UPLC–MS/MS method was set up for this purpose, acquiring mass spectra and adjusting mobile phase strength for commercially available standard solutions of MC-LR, MC-RR, MC-YR, and CYN. Then, matrix-matched calibration curves were prepared for each of the four cyanotoxins by directly spiking extracts of control fresh lettuce leaves with the desired concentrations of the multitoxin solution to obtain a linear range of 0.2–75 µg L^−1^, equivalent to 0.2–75 ng g^−1^ f.w. lettuce. To evaluate the efficiency of the proposed extraction and clean up methods, control fresh lettuce leaves (1.06 ± 0.05 g f.w.) were spiked with 1 mL of a multitoxin solution containing a mixture of the four cyanotoxins, at three concentration levels: 5, 20, and 50 µg L^−1^, leading to 5, 20, and 50 ng g^−1^ f.w. lettuce. Afterwards, leaves were lyophilized and toxins were extracted. For this purpose, 70% and 80% MeOH were assayed, considering the different percentages of MeOH (20–100%) used by other authors when extracting these toxins simultaneously from water samples [3,33,42], as no multitoxin methods are available in vegetables. As 80% MeOH yielded the best recovery results (data not shown), the studies continued with this MeOH concentration. Then, the lyophilized lettuce leaves (0.05 ± 0.002 g d.w.) were extracted with 6 mL of 80% MeOH, homogenized in an ultraturrax (1 min), sonicated (15 min), and stirred in an orbital shaker (15 min). The mixture was centrifuged (3700 rpm, 15 min) and the supernatant collected for the clean-up; a test was performed with the supernatant at different pH (7, 9, and 11), and pH 11 was selected because it yielded the best results (data not shown), allowing the neutralization and adsorption of the toxins, which are basic, to the sorbent material in the cartridge. The purification process was developed considering the methods described by Li et al. [4] and Zervou et al. [3]. The use of reverse phase C18 cartridges is suitable for the extraction of moderately polar organic compounds, such as MCs, from aqueous matrices. However, due to the hydrophilic nature of CYN, it cannot be extracted by SPE with C_18_ cartridges, but PGC cartridges have been successfully used. So, an assembly of a C_18_ Bakerbond^®^ cartridge (500 mg, 6 mL, Dicsa (Andalucía, Spain) and a BOND ELUT^®^ Carbon cartridge (Agilent Technologies, Amstelveen, The Netherlands)) was employed. Moreover, because CYN could not be retained in the C_18_ cartridge, this was set at the top and PGC at the bottom, and the order was reversed (PGC on top and C_18_ on bottom) for elution to avoid MCs retention in the PGC column. After adjusting the supernatant pH to 11, the following reagents were passed through the assembled cartridges: 6 mL DCM, 6 mL 100% MeOH, 6 mL H_2_O (pH 11), and sample (pH 11); then, cartridges were dried for 5 min and the order was inverted for elution of toxins with 10 mL DCM/MeOH (40/60) + 0.5% FA. The addition of DCM and FA to MeOH in the elution solvent is crucial to simultaneously extract CYN and MCs, due to their different polarities. Then, the extract was evaporated to dryness in a rotary evaporator and resuspended in 1 mL 20% MeOH for its analysis by UPLC-MS/MS. Three different percentages of MeOH were tested as redissolving solvent (20%, 50%, and 80% MeOH); 50% MeOH was rejected in the first place because it yielded the lowest recoveries for the four cyanotoxins; peak splitting was observed for CYN with 50% and 80% MeOH and its recovery was very low; so, finally, 20% MeOH was selected due to the best elution and recovery of the four cyanotoxins (data not shown).

### 4.3. Chromatographic Conditions

Chromatographic separation was performed using a UPLC Acquity (Waters) coupled to a Xevo TQ-S micro (Waters, Milford, MA, USA) consisting of a triple quadrupole mass spectrometer equipped with an electrospray ion source operated in positive mode. UPLC analyses were performed on a 100 × 2.1 mm XSelect HSS T3 2.5 µm column, at a flow rate of 0.45 mL min^−1^. A binary gradient consisting of (A) water and (B) acetonitrile, both containing 0.1% formic acid (*v*/*v*) was employed, and the injection volume was 5 µL. The elution profile was: 2% B (0.8 min), linear gradient to 70% B (6.2 min), 100% B (1 min), and, finally, 2% B (2 min). Multiple Reaction Monitoring (MRM) was applied, where the parent ions and fragments ions were monitored at Q1 and Q3, respectively. For UPLC-ESI-MS/MS analyses, the mass spectrometer was set to the following optimised tune parameters: Capillary voltage: 1.0 kV; source temperature: 500 °C; source desolvation gas flow: 1000 L/h; and source cone gas flow: 50 L h^−1^.

### 4.4. Analytical Criteria for Method Validation

For validation of the extraction and quantification method, several analytical parameters were calculated, such as linearity, sensitivity, precision, and recovery, considering the guidelines from Eurachem [51], and from González and Herrador [49], and the AOAC [50], For this purpose, three validation standards were employed for each cyanotoxin, performing the measures in triplicate each day for three consecutive days, covering the optimal working range. One mL solutions with three different cyantoxins concentrations (5, 20, and 50 µg L^−1^) were added to lettuce leaves to obtain 5, 20, and 50 ng g^−1^ f.w., respectively. Precision and recovery were obtained by applying a one-factor analysis of variance (ANOVA), as explained in the Results and Discussion section, and then the results were compared with the respective tabulated reference values for each toxin concentration level. Besides, a robustness assay was also conducted to evaluate the ability of the method to stay unaffected despite small variations inherent in the analytical procedure in some parameters tested with the Student’s t test, according to Youden's procedure (1967) [59]. This study was performed by spiking the leaf samples with an intermediate concentration of 20 µg L^−1^ multitoxin solution (MC-LR, MC-RR, MC-YR, and CYN) (equivalent to 20 ng g^−1^ f.w.), and the parameters were: (F1) Sonication time, (F2) stirring time, and (F3) time for the sample to pass through the cartridge. By combination of these parameters, eight different possibilities were assessed (Table 3). The weight of every factor is determined as the divergence of the medium results obtained at the level +1 and obtained at the level −1.

### 4.5. Exposure of Edible Vegetables Under Laboratory Conditions and Analysis of Toxins by the Validated Method

Plants of lettuce (*Lactuca sativa*) and spinach (*Spinacia oleracea*) were obtained from a local market (Porto, Portugal) as sprouts. Before their cultivation in a hydroponic system, all remaining soil was removed from the roots by washing with deionized water. Then, plants were introduced into opaque glass jars, ensuring that the roots were completely immersed in Jensen culture medium [60] at pH 6.5, as explained in Freitas et al. [61]. After an acclimation period of one week with white fluorescent light (14–10 h, light–dark period) and 21 ± 1 °C, plants were exposed in the medium to a solution containing MCs and CYN extracted from *M. aeruginosa* and *C. ovalisporum* cultures, respectively, at concentrations of 10 or 50 µg L^−1^ (*n* = 5 per condition assayed). The culture medium with the solution containing MCs and CYN was changed three times a week for 21 d. Five plants per species were not exposed to the toxins (control groups). After the 21-d exposure period, plants were washed with distilled water, frozen, and lyophilized (Telstar Lyoquest) for analysis of MCs and CYN in leaves and roots, following the validated method presented in this work.

The *M. aeruginosa* culture (LEGE 91094) and the *C. ovalisporum* culture (LEGE X-001), isolated from Lake Kinneret, Israel [62], were grown in Z8 medium in the Interdisciplinary Centre of Marine and Environmental Research, CIIMAR (Porto, Portugal) [63]. MCs and CYN extraction was performed by the methods of Pinheiro et al. [64] and Welker et al. [65], respectively. Analysis by HPLC-PDA showed 0.2 mg MC-LR g^−1^ at 9.75 min and 2.9 mg CYN g^−1^ at 6.305 min.

## Figures and Tables

**Table 1:** Estimations of within-day repeatability (S_w_), between-day repeatability (S_B_), intermediate precision (intra-laboratory reproducibility, S_IP_), and its relative standard deviation (%RSD_IP_) for MC-LR, MC-RR, MC-YR, and CYN, at three concentration levels, in three different days, assayed in lettuce samples. Limits of detection (LOD) and quantitation (LOQ) for the lettuce matrix. RSD_AOAC_ (%): 16–22% for 5 µg L^−1^ and 8–16% for 20 and 50 µg L^−1^. Acceptable Recovery Range (%) by AOAC: 40–115% for 5 µg L^−1^ and 60–110% for 20 and 50 µg L^−1^.

	Validation Parameters						
	Toxin Concentration Level (µg L^−1^)	S_W_	S_B_	S_IP_	RSD_IP_ (%)	LOD (ng g^−1^ f.w. ^1^)	LOQ (ng g^−1^ f.w. ^1^)
MC-LR	5	0.20	1.10	0.66	21.68	0.06	0.16
	20	2.06	2.20	2.11	13.21		
	50	4.62	8.85	6.35	15.12		
MC-RR	5	0.14	0.21	0.17	8.31	0.23	0.50
	20	1.38	2.36	1.77	9.54		
	50	3.30	8.47	5.58	12.58		
MC-YR	5	0.23	0.74	0.46	19.86	0.42	0.91
	20	1.32	1.79	1.49	11.14		
	50	2.67	4.93	3.59	9.64		
CYN	5	0.28	0.65	0.44	19.30	0.07	0.19
	20	0.82	1.20	0.96	6.92		
	50	1.65	5.85	3.64	11.62		

^1^ f.w.: Fresh weight. Microcystin-LR (MC-LR), MC-RR, MC-YR, and Cylindrospermopsin (CYN).

**Table 2:** Coding rules for combination of the parameters in the robustness study and *t* values obtained for each parameter after the significance *t* test was applied.

Combined Variables			Toxins	*t* Values
F1	High (+)	15 min	MC-LR	1.793
			MC-RR	0.232
	Low (−)	10 min	MC-YR	1.996
			CYN	1.241
F2	High (+)	15 min	MC-LR	0.059
			MC-RR	0.381
	Low (−)	10 min	MC-YR	0.042
			CYN	0.358
F3	High (+)	1 min and 15 s	MC-LR	0.346
			MC-RR	0.234
	Low (−)	1 min	MC-YR	1.055
			CYN	2.231

F1: Sonication time of the samples; F2: Stirring time of the samples; and F3: Time for the sample to pass through the cartridge.

**Table 3:** Possible combinations (C1–C8) of parameters for the robustness study.

Combination Possibilities	F1	F2	F3
C1 (+++)	15 min	15 min	1 min
C2 (++−)	15 min	15 min	1 min 15 s
C3 (+−+)	15 min	10 min	1 min
C4 (+−−)	15 min	10 min	1 min 15 s
C5 (−++)	10 min	15 min	1 min
C6 (−+−)	10 min	15 min	1 min 15 s
C7 (−−+)	10 min	10 min	1 min
C8 (−−−)	10 min	10 min	1 min 15 s

F1: Sonication time of the samples; F2: Stirring time of the samples; and F3: Time for the sample to pass through the cartridge.
